# Copy number variation in *DRC1* is the major cause of primary ciliary dyskinesia in the Japanese population

**DOI:** 10.1002/mgg3.1137

**Published:** 2020-01-20

**Authors:** Kazuhiko Takeuchi, Yifei Xu, Masako Kitano, Kazuki Chiyonobu, Miki Abo, Koji Ikegami, Satoru Ogawa, Makoto Ikejiri, Mitsuko Kondo, Shimpei Gotoh, Mizuho Nagao, Takao Fujisawa, Kaname Nakatani

**Affiliations:** ^1^ Department of Otorhinolaryngology Head & Neck Surgery Mie University Graduate School of Medicine Tsu Japan; ^2^ Department of Respiratory Medicine Kanazawa University Kanazawa Japan; ^3^ Department of Anatomy and Developmental Biology Graduate School of Biomedical and Health Sciences Hiroshima University Hiroshima Japan; ^4^ Electron Microscopy Research Center Mie University Graduate School of Medicine Tsu Japan; ^5^ Department of Central Laboratories Mie University Hospital Tsu Japan; ^6^ Department of Respiratory Medicine Tokyo Women's Medical University Tokyo Japan; ^7^ Department of Drug Discovery for Lung Diseases Graduate School of Medicine Kyoto University Kyoto Japan; ^8^ Institute for Clinical Research National Hospital Organization Mie National Hospital Tsu Japan; ^9^ Department of Genomic Medicine Mie University Hospital Tsu Japan

**Keywords:** copy number variation, *DRC1*, ultrastructure

## Abstract

**Background:**

Primary ciliary dyskinesia (PCD) is a rare genetic disorder caused by functional impairment of cilia throughout the body. The involvement of copy number variation (CNV) in the development of PCD is largely unknown.

**Methods:**

We examined 93 Japanese patients with clinically suspected PCD from 84 unrelated families. CNV was examined either by exome sequencing of a PCD gene panel or by whole‐exome sequencing (WES). The identified alterations were validated by PCR and Sanger sequencing. Nasal ciliary ultrastructure was examined by electron microscopy.

**Results:**

Analysis of CNV by the panel or WES revealed a biallelic deletion in the dynein regulatory complex subunit 1 (*DRC1*) gene in 21 patients, which accounted for 49% of the PCD patients in whom a disease‐causing gene was found. Sanger sequencing of the PCR product revealed a 27,748‐bp biallelic deletion including exons 1–4 of *DRC1* with identical breakpoints in all 21 patients. The ciliary ultrastructure of the patients with this CNV showed axonemal disorganization and the loss or gain of central microtubules.

**Conclusion:**

The deletion of *DRC1* is the major cause of PCD in Japan and this alteration can cause various ciliary ultrastructural abnormalities.

## Graphical abstract

Analysis of copy number variation revealed a biallelic deletion in the dynein regulatory complex subunit 1 (*DRC1*) gene in 21 patients, which accounted for 49% of the primary ciliary dyskinesia patients in whom a disease‐causing gene was found. Sanger sequencing of the PCR product revealed a 27,748‐bp biallelic deletion including exons 1–4 of *DRC1* with identical breakpoints in all 21 patients. The ciliary ultrastructure of the patients with this copy number variation showed axonemal disorganization and the loss or gain of central microtubules.

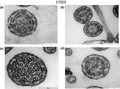

## 1 INTRODUCTION

Primary ciliary dyskinesia (PCD) is a rare genetic disorder of ciliary function that affects approximately 1 in 20,000 live births (Afzelius, Mossberg, & Bergstrom, 1995). PCD is inherited in an autosomal recessive (Afzelius et al., 1995) or X‐linked manner (Olcese et al., 2017; Paff et al., 2017). The symptoms of PCD are diverse, including *situs inversus*, chronic oto‐rhino‐pulmonary infections, and infertility, and can vary between patients. This heterogeneity makes the diagnosis of PCD challenging, particularly when *situs inversus* is absent and the other symptoms are mild (Sommer et al., 2011).

In order to identify disease‐causing gene mutations in PCD, we made a targeted next‐generation sequencing (NGS) panel of 32 PCD genes and examined Japanese patients suspected of PCD for single nucleotide mutations in these genes (Takeuchi et al., 2018). Although it has been reported that 21 PCD genes cover approximately 65% of mutations in PCD patients (Knowles, Daniels, Davis, Zariwala, & Leigh, 2013), our previous study revealed a mutation in only 10 of 46 patients (12.5%) in 7 families (Takeuchi et al., 2018). It is estimated that 15%–29% of PCD is caused by a mutation in *DNAH5* (Zariwala, Knowles, & Leigh, 2015). Since these data are based on European and American populations, it is possible that a different gene may be the major cause of PCD in Japan.

Copy number variations (CNVs) affecting protein‐coding genes contribute substantially to human diversity and disease (Ruderfer et al., 2016). However, CNV analysis in PCD has been very limited. Marshall et al. (2015) found clinically significant CNV in four families with PCD. We hypothesized that CNVs play an important role as the cause of PCD in the Japanese population. Therefore, in the present study, we analyzed CNV by NGS in 93 Japanese patients from 84 unrelated families suspected of PCD and found CNV in *DRC1* (*CCDC164*, OMIM 615288) in 17 families. Moreover, we examined the ultrastructure of cilia in those patients. Unlike studies reporting normal ciliary ultrastructure in subjects with *DRC1* mutations (Shapiro & Leigh, 2017; Wirschell et al., 2013), we found particular ultrastructural defects in patients with CNV in *DRC1* by transmission electron microscopy (TEM).

## 2 METHODS

### 2.1 Ethical compliance

The study was performed in accordance with the principles expressed in the Declaration of Helsinki and was approved by Mie University Ethics Committee (No. 1363). Written informed consent was obtained from all subjects or their guardians. The methods were performed in accordance with the relevant guidelines and regulations.

### 2.2 Patients and methods

The subjects consisted of 93 patients (40 males and 53 females; age range, 1 month–67 years) who were clinically suspected of PCD from 84 families. Genomic DNA was extracted from peripheral blood samples taken from the forearm of each participant. DNA samples were obtained from probands as well as other members of the family whenever available. The patients have been referred from many areas of Japan's main island and Kyushu island (Figure S1).

The diagnosis of PCD requires the presence of the characteristic clinical phenotype, such as chronic rhinosinusitis, otitis media with effusion, and bronchiectasis, and either (a) specific ciliary ultrastructural defects identified by TEM in biopsy samples of the respiratory epithelium or (b) a mutation in one of the genes known to be associated with PCD (Zariwala et al., 2015). We usually perform TEM and genetic analysis; however, some blood samples were sent from other facilities without TEM having been performed.

For CNV analysis, we utilized our NGS panel of 32 PCD genes (Takeuchi et al., 2018) or whole‐exome sequencing (Kano et al., 2016). For the NGS panel, we designed primers using Ion AmpliSeq Designer (Thermo Fisher Scientific, Inc.) to screen 32 known PCD genes (*ARMC4*, *C21orf59*, *CCDC103*, *CCDC114*, *CCDC151*, *CCDC39*, *CCDC40*, *CCDC65*, *CCNO*, *DNAAF1*, *DNAAF2*, *DNAAF3*, *DNAAF5*, *DNAH1*, *DNAH11*, *DNAH5*, *DNAH8*, *DNAI1*, *DNAI2*, *DNAL1*, *DRC1*, *DYX1C1*, *HYDIN*, *LRRC6*, *MCIDAS*, *NME8*, *RSPH1*, *RSPH3*, *RSPH4A*, *RSPH9*, *SPAG1*, and *ZMYND10*). Variant annotation was performed with Ion Reporter Version 5.0 (Life Technologies) using data integrated from a variety of public databases. One of the main sources of information for calling CNV segments is read coverage, which reflects the abundance of any given reference DNA sequence in the sample.

For whole‐exome sequencing, proband DNA was amplified with an Ion AmpliSeq™ Exome RDY Kit (Life Technologies; Thermo Fisher Scientific, Inc.). The samples were sequenced with a Proton PI Chip version 3 and the Ion Proton Semiconductor Sequencer System (Life Technologies; Thermo Fisher Scientific, Inc.). Base calling, preprocessing of the reads, short read alignment, and variant calling were performed with Torrent Suite, Torrent Variant Caller (version 5.0; Thermo Fisher Scientific, Inc.), and the default parameters recommended for the AmpliSeq Exome Panel (low stringency calling of germline variants, version, April 2014). Variant annotation was performed with Ion Reporter, version 5.0 (Life Technologies; Thermo Fisher Scientific, Inc.).

Alterations identified by NGS were validated via PCR and Sanger sequencing with a 3500 Series Genetic Analyzer (Thermo Fisher Scientific, Inc.).

Nasal and exhaled nitric oxide (NO) levels were measured via an ANALYZER CLD 88^®^ (ECO MEDICS AG) according to the recommendations of the American Thoracic Society and European Respiratory Society (2005).

Nasal mucosa was obtained for electron microscopy. Under local anesthesia, a small amount of nasal mucosa was extracted from the patient's inferior turbinate. More than 10 cilia from each patient were examined via TEM (JEM‐1011; JEOL).

## 3 RESULTS

By NGS analysis, significant CNV in *DRC1*, with high confidence (>40) and precision (>50) scores, was called in 21 patients from 17 families (Table 1). All 21 patients had deletions in both copies of *DRC1*, with the length of the predicted deletion ranging from 22,561 to 22,582 bp, with a mean of 22,575 bp.

**Table 1:** Calls on *DRC1* CNV by NGS

Family	Patient	Sex	Age (years)	Length (bp)	Cytoband	Confidence	Precision	
1	16162 (III‐1)	F	25	22,582	2p23.3 (26624799–26647381) × 0	75.8799	83.5775	†
	17002 (III‐2)	M	23	22,582	2p23.3 (26624799–26647381) × 0	75.9125	83.61	†
	16163 (III‐3)	F	20	22,582	2p23.3 (26624799–26647381) × 0	75.9125	83.61	†
2	4D4C4733 (III‐1)	F	16	22,582	2p23.3 (26624799–26647381) × 0	75.9125	83.61	†
3	17B630C2	M	5	22,582	2p23.3 (26624799–26647381) × 0	75.8953	68.1978	†
	342E9543	M	1	22,582	2p23.3 (26624799–26647381) × 0	75.7308	83.4283	†
4	J03	F	64	22,561	2p23.3 (26624795–26647356) × 0	44.7066	60.0954	^‡^
	J04	F	61	22,561	2p23.3 (26624795–26647356) × 0	44.7067	60.096	^‡^
5	0D389546	F	18	22,561	2p23.3 (26624795–26647356) × 0	44.4337	58.5747	^‡^
6	1BD3A017	M	42	22,561	2p23.3 (26624795–26647356) × 0	44.7067	60.0958	^‡^
7	16039	F	32	22,561	2p23.3 (26624795–26647356) × 0	44.7061	60.0936	^‡^
8	16052	F	12	22,561	2p23.3 (26624795–26647356) × 0	44.7072	60.098	^‡^
9	AEB51AF4	F	7	22,561	2p23.3 (26624795–26647356) × 0	44.7061	60.0962	^‡^
10	16149	F	54	22,582	2p23.3 (26624799–26647381) × 0	75.9125	83.61	†
11	17028	F	3m	22,582	2p23.3 (26624799–26647381) × 0	75.9125	83.61	†
12	17059	M	21	22,582	2p23.3 (26624799–26647381) × 0	75.9125	83.61	†
13	17061	F	11	22,582	2p23.3 (26624799–26647381) × 0	75.9125	83.61	†
14	18003	F	12	22,582	2p23.3 (26624799–26647381) × 0	68.3116	76.0091	†
15	18046	F	11	22,582	2p23.3 (26624799–26647381) × 0	75.9125	83.61	†
16	18047	M	44	22,582	2p23.3 (26624799–26647381) × 0	75.9125	83.61	†
17	18097	F	25	22,582	2p23.3 (26624799–26647381) × 0	75.9125	83.61	†

Note

Confidence is the log likelihood that the called copy number state is not normal ploidy, that is, two on autosomes (reflects the likelihood of the region's ploidy number being different from the normal ploidy of 2).

Precision is the log likelihood that the called copy number state is different from the next closest copy number state (reflects the likelihood that the precise ploidy number is correct). Analyses were performed using either the PCD gene panel (†) or whole‐exome sequencing (‡).

**Figure 1:**
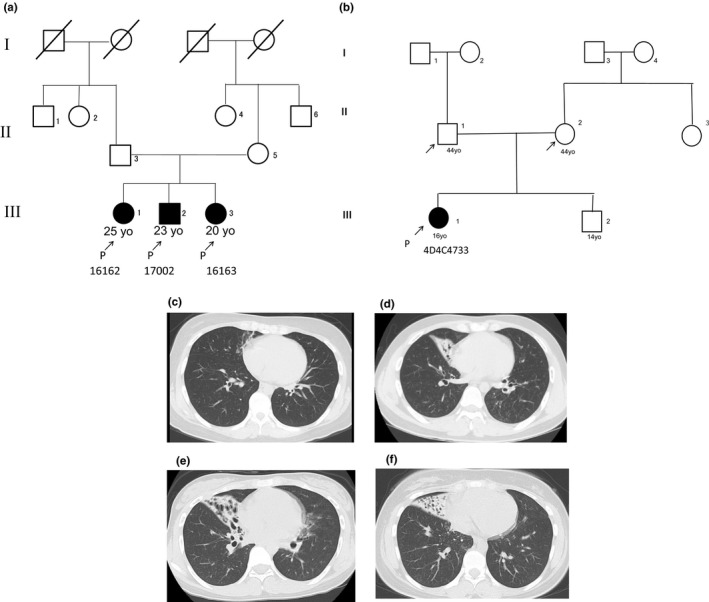
Clinical information of the two families. The pedigree of Family 1 and Family 2 is shown, respectively (a, b). Three siblings in Family 1 presented with similar clinical symptoms. Chest computed tomography scans in Family 1: III‐1 (c), III‐2 (d), and III‐3 (e) and in Family 2: III‐1 (f)

Initially, we performed detailed analysis of two families. In Family 1 (Figure 1a), all three siblings (III‐1–3) had very similar clinical courses. They developed rhinorrhea, nasal obstruction, and productive cough when they were small children. Chronic rhinosinusitis was observed and abnormalities were found on chest X‐rays and chest computed tomography (Figure 1c–e) in the three siblings. None of them had *situs inversus*. Erythromycin was started in III‐1 and III‐3.

In Family 2 (Figure 1b), a 16‐year‐old female (III‐1) was referred to our clinic with a chief complaint of persistent posterior rhinorrhea. Otitis media with effusion were detected when she was aged 1 year. Anterior and posterior rhinorrhea started when she was a small child, and productive cough developed when she was a junior high school student. Nasal NO production was 67 nl/min, which is low and compatible with PCD (Leigh et al., 2013). Chest computed tomography showed an infiltrative shadow in her right middle lobe (Figure 1f), but she did not have *situs inversus*.

**Figure 2:**
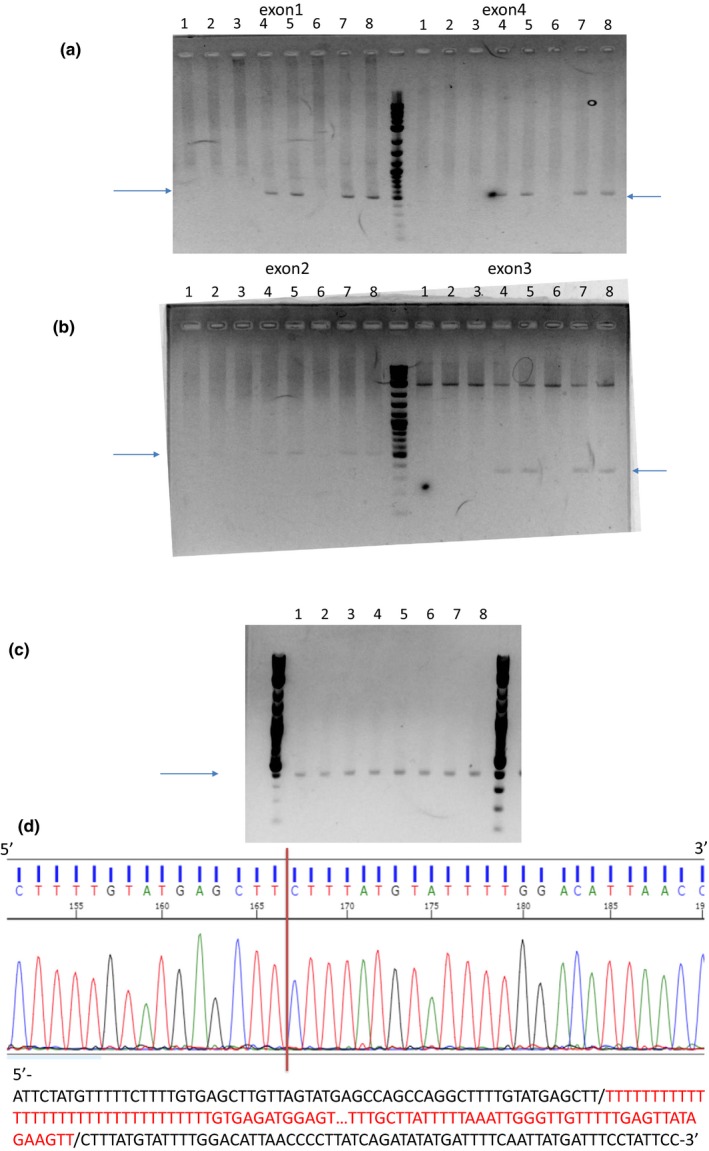
The results of PCR to amplify exons 1 and 4 (a) and exons 2 and 3 (b) are shown. Four sets of PCR primers specific for exons 1, 2, 3, and 4 were used, respectively. Bands with the expected sizes were observed only in the parents, but not in the four probands. Lanes 1–5: III‐1, III‐2, III‐3, II‐3, and II‐5 of Family 1. Lanes 6–8; III‐1, II‐1, and II‐2 of Family 2. The results of PCR to amplify the region including the whole deleted length (exons 1–4) (c). Bands with the expected sizes were observed in all subjects examined (c). Lanes 1–5: III‐1, III‐2, III‐3, II‐3, and II‐5 of Family 1. Lanes 6–8; III‐1, II‐1, and II‐2 of Family 2 (c). Sanger sequencing of the PCR products revealed a 27,748‐bp deletion with the breakpoints shown with a vertical red bar (d). The deleted sequences are shown in red below

We designed four sets of PCR primers specific for exons 1, 2, 3, and 4 of *DRC1*, respectively (Table S1) and examined whether each exon could be amplified. The bands with expected sizes (513, 515, 357, and 521 bp, for exons 1, 2, 3, and 4, respectively) could be observed only in the parents (II‐3 and II‐5 of Family 1 and II‐1 and II‐2 of Family 2), but not in the four probands (III‐1, III‐2, and III‐3 of Family 1 and III‐1 of Family 2) (Figure 2a,b). Sequencing of PCR products of the four exons in the parents did not reveal any deletions.

Next, we designed PCR primers to identify the deletion breakpoints so that PCR produced a band of approximately 400 bp when exons 1–4 were deleted (Table S1). Bands with the expected size were obtained in the four probands and four parents (Figure 2c). No bands were observed when normal subjects without the *DRC1* CNV were tested. Sanger sequencing of the PCR products revealed a 27,748‐bp deletion with the breakpoints shown (NC_000002.11:g.[26,620,908_26,648,655del];[26,620,908_26,648,655del]) in all four probands and four parents (Figure 2d).

We performed PCR to clarify the length of the deletion in the remaining 17 patients from 15 families shown to have a deletion of both copies of *DRC1* by NGS. All of the patients showed bands of the expected length (~400 bp). Sanger sequencing of the PCR products showed the same breakpoints mentioned above in all 17 patients from 15 families. Details of all the variants detected in 43 PCD patients from 35 families are shown (Table S2). The geographical distribution of *DRC1* families was not different from that of non‐*DRC1* families (Figure S1).

The 21 patients from 17 families with this *DRC1* CNV (Table 2) consisted of 6 males and 15 females and their ages ranged from 3 months to 64 years. None had *situs inversus*. Measurement of nasal NO production was possible only in six patients; NO production was extremely low in these patients.

**Table 2:** Prevalence of gene variations in the Japanese PCD patients

Gene	Number of families (patients)	Percentage^a^	Number of patients with *situs inversus*
*DRC1*	17 (21)	49%	0
*DNAH5*	11 (14)	31%	8
*DNAH11*	4 (4)	12%	1
*DNAI1*	1 (2)	3%	0
*CCDC40*	1 (1)	3%	0
*RSPH4A*	1 (1)	3%	0
Total	35 (43)	100%	9

a The percentage is calculated based on the number of families.

In 11 of the 21 *DRC1* CNV patients, TEM was available. Among all of these patients, except one (#18003), certain structural abnormalities were found (Table 3). The percentage of cilia that were abnormal in each patient is also shown (Table 3).

**Table 3:** Clinical profiles of the patients with *DRC1* CNV

Family	Patient	Sex	Age at diagnosis (years)	TEM^a^	*Situs inversus*	Nasal NO production (nL/min)
1	16162 (III‐1)	F	25	MTD (7.7%)	*N*	NA
	17002 (III‐2)	M	23	MTD (25%)	*N*	NA
	16163 (III‐3)	F	20	MTD (7.8%)	*N*	NA
2	4D4C4733 (III‐1)	F	16	MTD (23%)	*N*	67
3	17B630C2	M	5	CA (16%) MTD (9.3%)	*N*	3.3
	342E9543	M	1	CA (17%) MTD (67%)	*N*	3.3
4	J03	F	64	NA	*N*	NA
	J04	F	61	NA	*N*	NA
5	0D389546	F	18	NA	*N*	14.0
6	1BD3A017	M	42	CA (42%) MTD (11%)	*N*	NA
7	16039	F	32	NA	*N*	NA
8	16052	F	12	NA	*N*	NA
9	AEB51AF4	F	7	NA	*N*	NA
10	16149	F	54	NA	*N*	NA
11	17028	F	3m	NA	*N*	NA
12	17059	M	21	CA (8.3%) MTD (29%)	*N*	16.5
13	17061	F	11	CA (5.7%)	*N*	16.8
14	18003	F	12	Normal	*N*	NA
15	18046	F	11	NA	*N*	NA
16	18047	M	44	NA	*N*	NA
17	18097	F	25	CA (7.7%) MTD (54%)	*N*	NA

Abbreviations: CA, central apparatus abnormalities; MTD, microtubule disorganization; *N*, no; NA, not available; TEM, transmission electron microscopy.

a The percentage of abnormal cilia with CA or MTD is calculated against all of the observed cilia cross‐sections.

**Figure 3:**
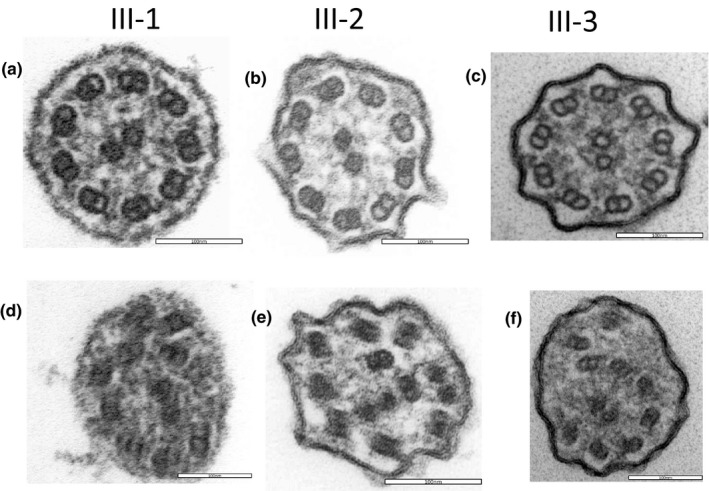
Electron microscopy of the three probands in Family 1 (a, d for III‐1; b, e for III‐2; c, f for III‐3). Most of the cilia were normal (a, b, c), but axonemal microtubular disorganization (d, e, f) was observed in some of the cilia in all 3 siblings

**Figure 4:**
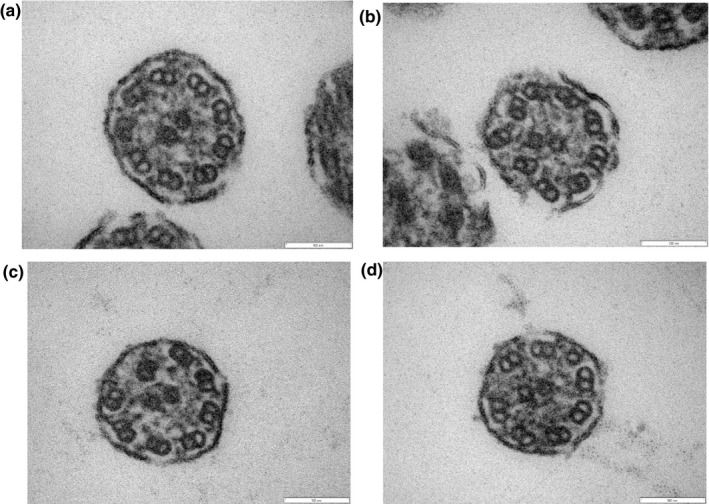
Electron microscopy of the proband in Family 2 (III‐1, #4D4C4733). Although approximately 80% of the cilia were normal (a), the remaining 20% had defects, mainly involving transposition of peripheral microtubule pairs (b, c) and singlets of peripheral microtubules (d)

In Family 1, most of the cilia were normal (Figure 3a–c), but axonemal microtubular disorganization (Figure 3d–f) was observed in some of the cilia in all three siblings. In Family 2, although approximately 80% of the cilia were normal (Figure 4a), the remaining 20% had defects, mainly involving the transposition of peripheral microtubule pairs (Figure 4b,c) and singlets of peripheral microtubules (Figure 4d).

**Figure 5:**
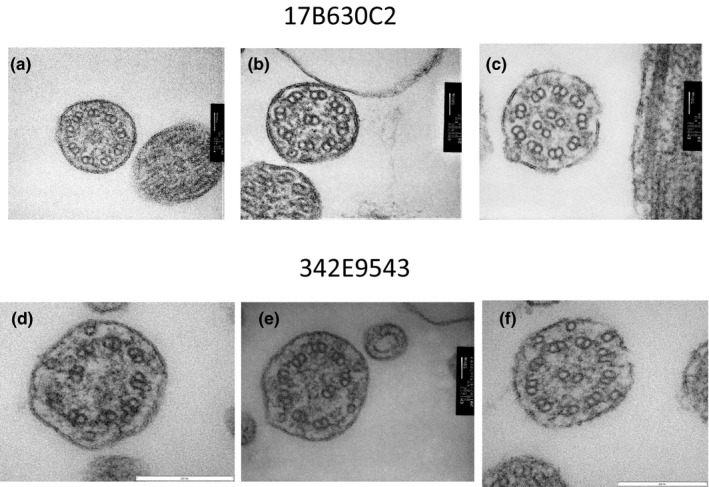
Electron microscopy of the two siblings in Family 3 (a, b, c for #17B630C2; d, e, f for #342E9543). In #17B630C2, three different abnormalities of central microtubules were observed: no central microtubules (a), one peripheral microtubule doublet transposed to the center of the axoneme with eight pairs of peripheral microtubules and no central microtubules (b), and two pairs of central microtubules (c). In #342E9543, peripheral microtubules were disarranged with no central microtubules (d, e) and numerous microtubule singlets were observed (f)

In a sibling of Family 3 (#17B630C2), the following three abnormalities of central microtubules were observed: no central microtubules (Figure 5a), one peripheral microtubule doublet transposed to the center of the axoneme with eight pairs of peripheral microtubules and no central microtubules (Figure 5b), and two pairs of central microtubules (Figure 5c). In the other sibling of Family 3 (#342E9543), peripheral microtubules were disarranged with no central microtubules (Figure 5d,e) and numerous microtubule singlets were observed (Figure 5f).

**Figure 6:**
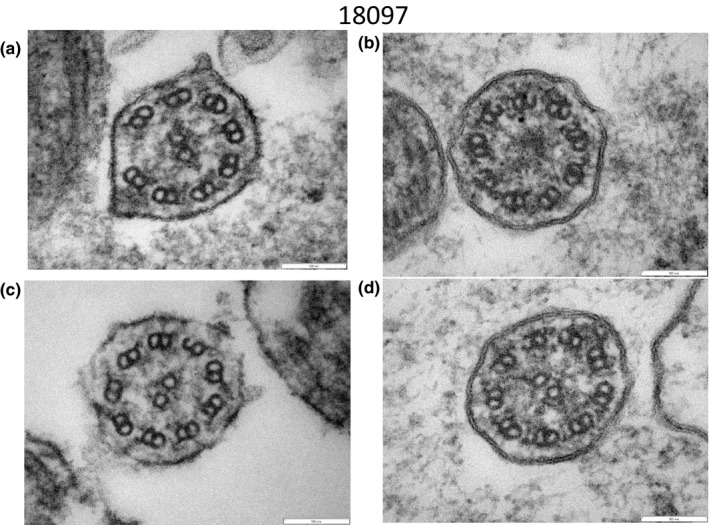
Electron microscopy of #18097. A pair of peripheral microtubules was absent (a). No central microtubules were observed (b). Ruptured B microtubules were observed (b, c, d)

**Figure 7:**
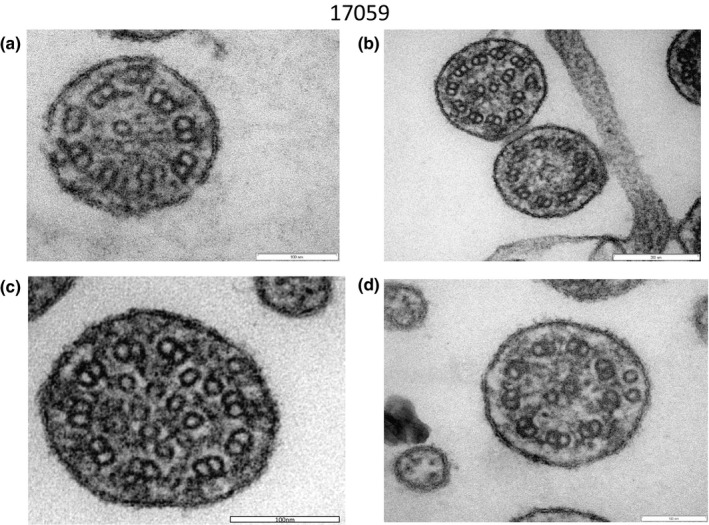
Electron microscopy of #17059. Peripheral microtubular disorganization was prominent (a, b). Central microtubules were lacking (b) or increased in number (c, d)

**Figure 8:**
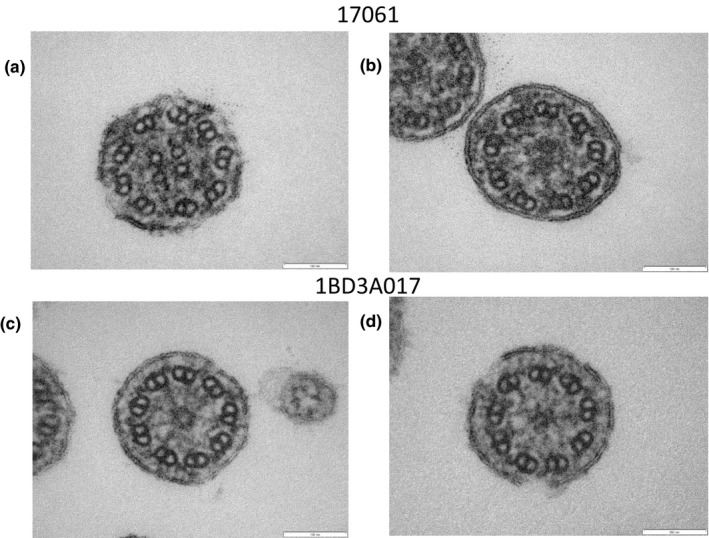
Electron microscopy of #17061 (a, b) and #1BD3A017 (c, d). The cilium had an additional pair of central microtubules (a). Some cilia were lacking central microtubules (b, c, d) and condensation was observed in the center of the cilia (c, d)

A variety of structural abnormalities was found in the remaining four patients (#18097, #17059, #17061 and #1BD3A017) (Figures 6, 7, 8): the absence of a pair of peripheral microtubules (Figure 6a), no central microtubules (Figure 6b), and ruptured B microtubules (Figure 6b–d). Peripheral microtubular disorganization was prominent (Figure 7a,b). Central microtubules were absent (Figure 7b) or increased in number (Figure 7c,d). The cilia had an additional pair of central microtubules (Figure 8a). Some cilia were lacking central microtubules (Figure 8b–d) and condensation was observed in the center of the cilia (Figure 8c,d).

## 4 DISCUSSION

DRC1 is a subunit of the nexin‐dynein regulatory complex (N‐DRC), an axonemal structure critical for the regulation of dynein motors (Wirschell et al., 2013). The present biallelic deletion of *DRC1* spans 27,748 bp, including exons 1–4. The deletion starts at 26,620,908 and ends at 26,648,655 on chromosome 2 (NC_000002.11) in GRCh37 [hg19], which is different by only a few base pairs from esv2657536 (NC_000002.12; 26,398,037_26,425,785del in GRCh38.p12 or NC_000002.11; 26,620,905_26,648,653del in GRCh37 [hg19]) (https://www.ncbi.nlm.nih.gov/dbvar/variants/esv2657536/). A recent report of a large deletion in *DRC1* (NM_145038.4:c.1‐3952_540+1331del) identified as causing PCD in two Asian patients (Morimoto et al., 2019) supports our findings. Thus, this CNV is likely relevant not only to Japanese but also to non‐Japanese Asians.

Among 84 families, we found this deletion in 17. Since we have identified disease‐causing mutations other than *DRC1* in 18 families in this group (Kano et al., 2016; Orimo et al., 2019; Takeuchi et al., 2018; Tanaka et al., 2012), *DRC1* acts as a disease‐causing gene in 49% of these Japanese PCD patients, followed by *DNAH5* (31%) and *DNAH11* (12%) (Table 2). The high prevalence of *DRC1* may be caused by the high frequency of this deletion in the Japanese population and non‐Japanese Asians. The minor allele frequency of this deletion in the Invitae cohort was calculated to be 0.31% (6/1,930) in Asians and 0.012% (6/49,832) in Caucasians (Morimoto et al., 2019).

*DRC1* is not a prevalent cause of PCD in non‐Asian populations. According to Knowles et al. (2013), the common genes causing PCD were *DNAH5* (15%–21%), *DNAI1* (2%–9%), *DNAAF1* (LRRC50) (4%–5%), *CCDC39* (2%–10%), *CCDC40* (2%–8%), *DNAH11* (6%), and *LRRC6* (3%). The percentage of individuals with biallelic *DRC1* mutations is not available. In Genereview (Zariwala et al., 2015), *DRC1* was classified as an uncommon genetic cause of PCD, but its exact prevalence was not reported.

Another important finding in this study was that this large deletion can cause various ciliary ultrastructural abnormalities. Previous studies reported that mutations in *DRC1* produce normal ciliary ultrastructure (Shapiro & Leigh, 2017; Wirschell et al., 2013) or at least an absence of nexin links (Wirschell et al., 2013). The structural abnormalities we found were various and different between the patients, but they can be summarized as central apparatus abnormalities and axonemal microtubular disorganization.

DRC1 is a central subunit for the assembly of the N‐DRC, which forms a cross‐bridge between the peripheral doublet microtubules of the axoneme and plays a crucial role in the regulation of ciliary beating (Heuser, Raytchev, Krell, Porter, & Nicastro, 2009). The N‐DRC has a role in the assembly and regulation of specific classes of inner dynein arm motors and may also restrict dynein‐driven microtubule sliding, thus aiding the generation of ciliary bending (Wirschell et al., 2013).

The observed abnormalities of the central apparatus are similar to the phenotypes of PCD mutations in radial spoke (RS)‐building proteins (Daniels et al., 2013; Kott et al., 2013; Lin et al., 2014). The connection between the N‐DRC and RSs could explain the defects in the central apparatus. The N‐DRC appears to connect to the base of a radial spoke, RS2 (Lin, Heuser, Carbajal‐González, Song, & Nicastro, 2012). During interphase, a filament composed of DRC1, DRC2, and DRC4 appears to bind to RS2 (Song et al., 2018). DRC2 is reportedly crucial for stabilizing RSs (Bower et al., 2018). Thus, the absence of DRC1 in the patients could loosen the attachment of RS to the axoneme, resulting in central apparatus abnormalities that are similar to those observed with RS mutations.

Heuser et al. (2009) divided the N‐DRC into two regions, a “linker” domain that binds to the B‐tubule of the peripheral doublet, and a “base plate” domain that binds to the A‐tubule. Oda, Yanagisawa, and Kikkawa (2015) described the detailed structure and location of DRC1, DRC2, and DRC4 proteins in the N‐DRC. These components start from the linker domain, span across the base plate, and end near the hole on the inner junction, to form a central scaffold for the N‐DRC. Taken together, the loss of function of DRC1 may weaken the N‐DRC and its attachment to the peripheral microtubules, leading to axonemal microtubule disorganization.

*Situs inversus* was not observed in any of the 21 patients with the *DRC1* deletion. Among the 43 patients in which disease‐causing genes were identified, only nine (21%) had *situs inversus* (Table 2). The general rule that approximately 50% of patients with PCD have *situs inversus* cannot be applied to the Japanese population. The absence of *situs inversus* is reasonable, because nodal cilia, which underlie the determination of left‐right laterality, lack the central apparatus and radial spokes (Hirokawa, Tanaka, Okada, & Takeda, 2006).

Lung disease and lung function are reportedly worse (Davis et al., 2015, 2019) in those with inner dynein arm and central apparatus defects with microtubular disorganization ultrastructural defects, most of whom have biallelic mutations in *CCDC39* or *CCDC40* (Antony et al., 2013). The limitation of this study is the lack of ciliary motility (waveform) measurement and lack of clinical data on lung function or respiratory microbiology. The clinical features including lung function as well as ciliary motility affected by the *DRC1* CNV should be studied in the future.

## 5 CONCLUSION

The deletion of *DRC1* is a major cause of PCD in Japan, and this alteration can cause various ciliary ultrastructural abnormalities.

## CONFLICT OF INTEREST

The authors have no conflict of interest to declare.
