# Supplementary material for: Copy number variation in DRC1 is the major cause of primary ciliary dyskinesia in the Japanese population
Source: Mol Genet Genomic Med. 2020 Jan 20;8(3):e1137. doi: 10.1002/mgg3.1137 (PMC7057087; doi:10.1002/mgg3.1137)
Supplement: Supplementary file 1 [file MGG3-8-e1137-s001.pdf]

Figure legend

Supplementary Figure 1: Distribution of PCD families with *DRC1* CNV (red dots) and those with variants other than *DRC1* CNV (black dots).

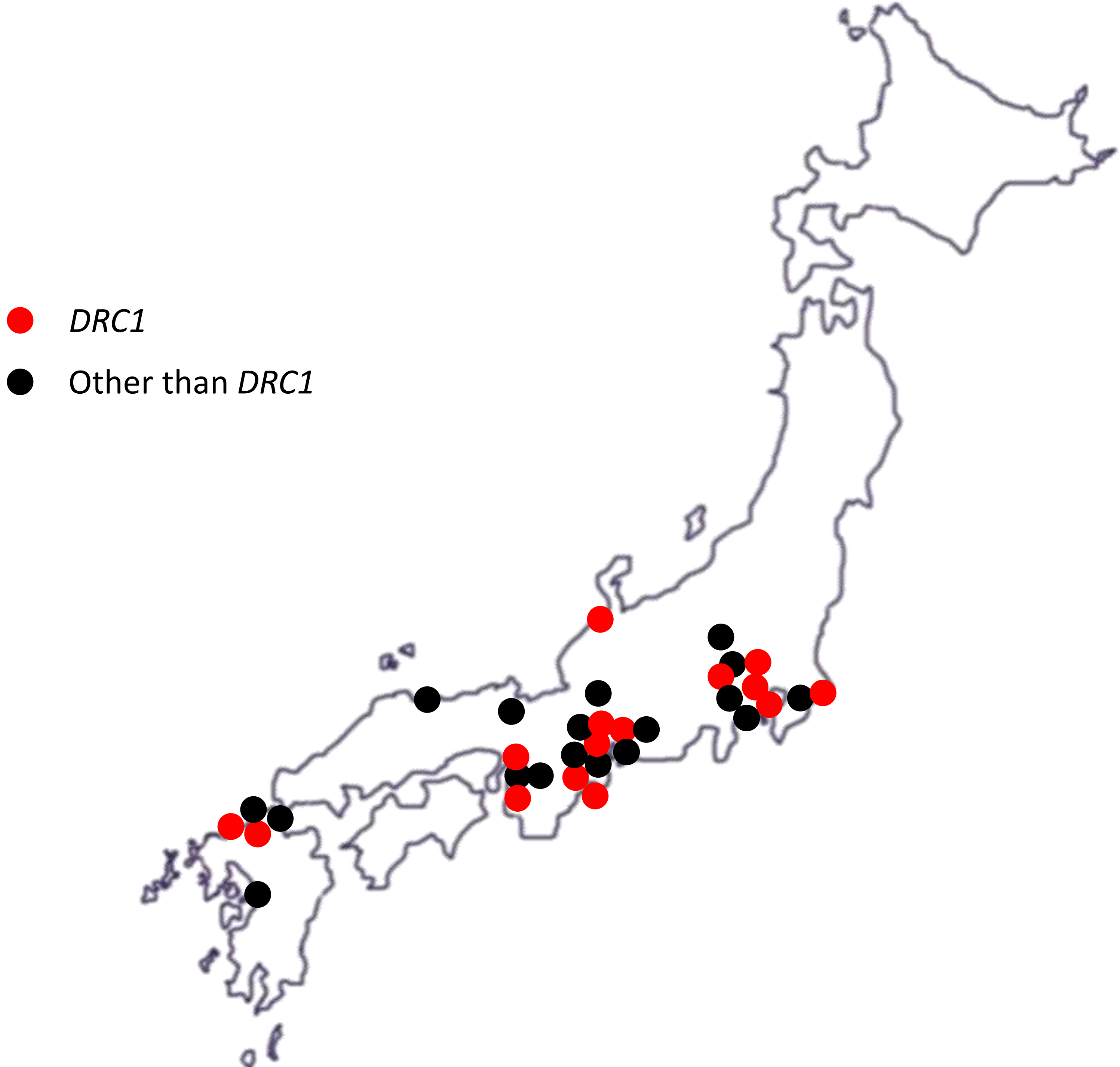
DRC1
Other than *DRC1*
